# Mangrove selective logging sustains biomass carbon recovery, soil carbon, and sediment

**DOI:** 10.1038/s41598-021-91502-x

**Published:** 2021-06-10

**Authors:** Daniel Murdiyarso, Sigit D. Sasmito, Mériadec Sillanpää, Richard MacKenzie, David Gaveau

**Affiliations:** 1 Center for International Forestry Research, Jl. CIFOR, Situgede, Bogor 16115 Indonesia; 2 Department of Geophysics and Meteorology, IPB University, Bogor, 16680 Indonesia; 3 NUS Environmental Research Institute, National University of Singapore, 21 Lower Kent Ridge Road, Singapore, 119077 Singapore; 4 Green Forest Product & Tech. Pte. Ltd., 3 Shenton Way, Singapore, 068805 Singapore; 5 Department of Geography, National University of Singapore, 1 Arts Link, Singapore, 117570 Singapore; 6 USDA Forest Service, Pacific Southwest Research Center, Institute of Pacific Islands Forestry, 60 Nowelo St., Hilo, HI 96720 USA; 7 TheTreeMap, Bagadou Bas, 46600 Martel, France

**Keywords:** Climate-change ecology, Ecosystem services, Forest ecology, Wetlands ecology, Climate-change ecology, Forest ecology, Wetlands ecology, Ecology, Climate sciences, Environmental sciences

## Abstract

West Papua’s Bintuni Bay is Indonesia’s largest contiguous mangrove block, only second to the world’s largest mangrove in the Sundarbans, Bangladesh. As almost 40% of these mangroves are designated production forest, we assessed the effects of commercial logging on forest structure, biomass recovery, and soil carbon stocks and burial in five-year intervals, up to 25 years post-harvest. Through remote sensing and field surveys, we found that canopy structure and species diversity were gradually enhanced following biomass recovery. Carbon pools preserved in soil were supported by similar rates of carbon burial before and after logging. Our results show that mangrove forest management maintained between 70 and 75% of the total ecosystem carbon stocks, and 15–20% returned to the ecosystem after 15–25 years. This analysis suggests that mangroves managed through selective logging provide an opportunity for coastal nature-based climate solutions, while provisioning other ecosystem services, including wood and wood products.

## Introduction

Indonesia’s mangroves occupy nearly 25% of the world’s mangrove area, representing 3.14 Pg of carbon stocks that can potentially contribute to global climate change mitigation^1,2^. However, land-use and land-cover changes (LULCCs) have generated significant losses for these coastal forests over the past few decades^3^. Such LULCC activities include mangrove conversion to aquaculture and oil palm plantation^3^, as well as timber harvesting as part of forest management activities^4^. Nationally, mangrove clearance rates averaged 52,000 ha every year in 1980–2005^1^.

Since the 1980s, more than 100,000 ha of Indonesia’s mangroves have been under forest management; with 80% of these situated in West Papua, it represents the world’s largest mangrove forest management site^4,5^. Unlike mangrove conversion, which commonly leads to substantial direct carbon stock loss from all pools^6^, the impact of forest management on soil carbon pool conservation remains debatable^7–9^. Soil carbon pools share 85% of the total ecosystem carbon stocks (TECS) in mangrove ecosystems^10^; its magnitude relying on the depth of the organic layer and rates of carbon burial facilitated by sedimentation.

The 2013 IPCC Supplement to the 2006 IPCC Guidelines^11^ suggests that, up to a depth of 1 m, forest management generates zero change in soil carbon pools, despite carbon fluxes being altered by reduced carbon burial^9^, and a potential increase in decomposition^12^. While a soil mass balance approach across a detailed depth profile found no evidence of soil carbon stocks being lost after forest management^8^, further investigation into carbon burial trends before and after logging is critical to resolve uncertainty^9,13^. Such information is particularly important in attempts to include mangrove in greenhouse gas (GHG) emission reduction strategies.

**Figure 1:**
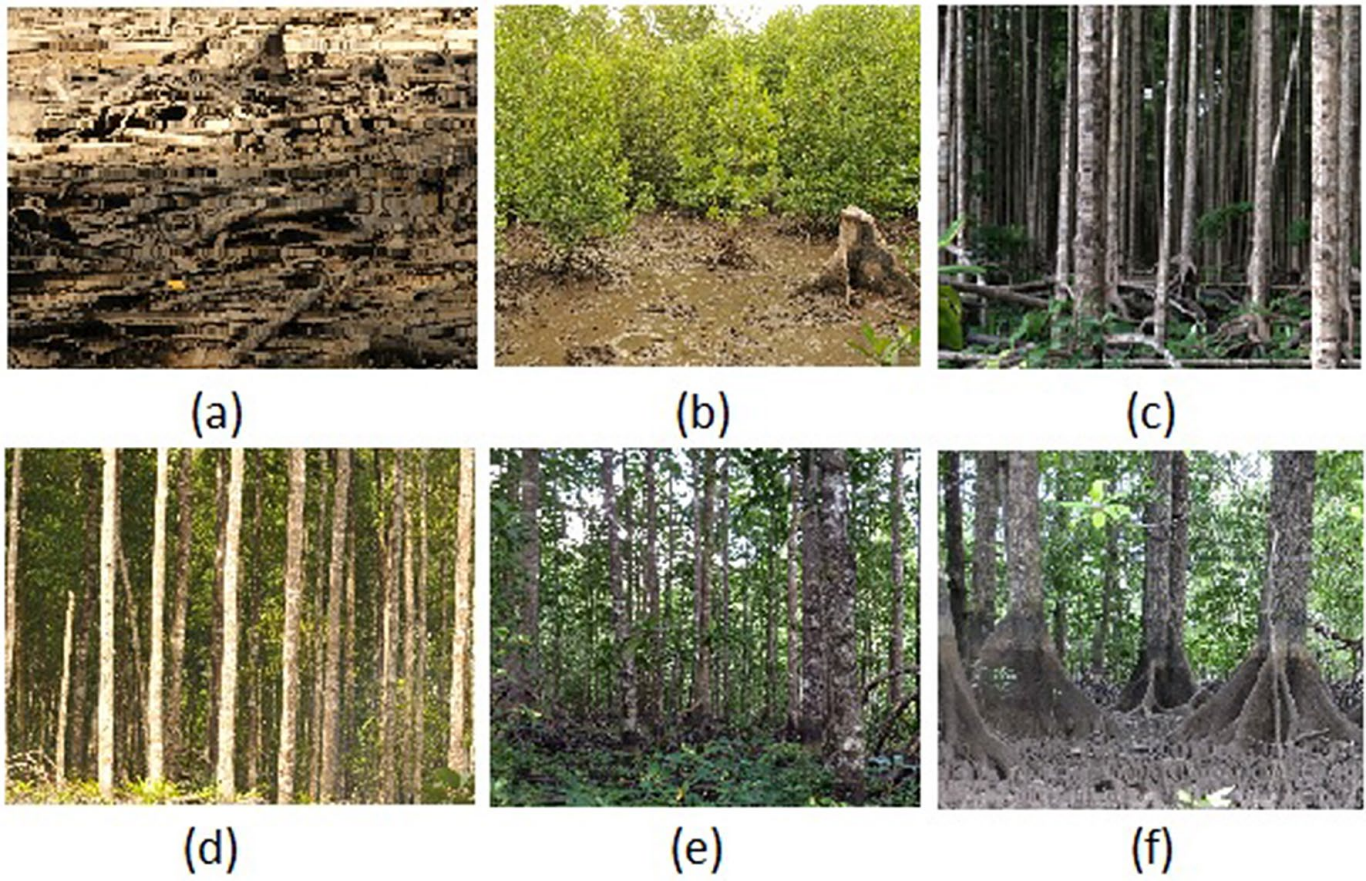
General conditions of mangrove stand at: (**a**) 0 years, (**b**) 5 years, (**c**) 10 years, (**d**) 15 years, and (**e**) 25 years post-harvest; and (**f**) in protected or unharvested mangroves.

Here, we evaluate the effects of selective logging-for-timber on mangrove blue carbon storage deliverable capacity, in a 82,120 ha forest management concession with a 30-year harvest rotation period^5^. Specifically, we assessed forest structure, species diversity, TECS, extracted timber volume and area, sediment accretion and carbon burial across sites harvested for timber, following regeneration after 0, 5, 10, 15 and 25 years, and in protected control forests (Fig. 1).

## Results and discussion

Our analysis suggests that, over 465 ha of mangrove area, almost 83% of aboveground tree biomass were harvested annually for commercial timber purposes, using a keyhole harvest pattern (Fig. S3b). Yet after 25 years of natural and human-induced regeneration, both field- and satellite-based assessments reveal that biomass carbon stocks and canopy cover had fully recovered. Our approach using space-for-time substitution indicates that manual selective logging did not significantly affect soil carbon stocks and rates of annual carbon burial. While the differences in soil carbon stock between sites may be due to the diverse hydro-geomorphic settings^8,14^ the mangrove root mass in the top 1-m were not disturbed by manual logging activities. Similar situation was found in Tampa Bay, Florida where peat formation from root mass has enhance carbon sequestration^15^. These findings reduce uncertainty around the effects of mangrove forest management on the long-term functional capacity of blue carbon storage and provide evidence that managed mangrove ecosystems may deliver nature-based climate solutions.

### Recovery of forest structure, canopy cover and species diversity

**Figure 2:**
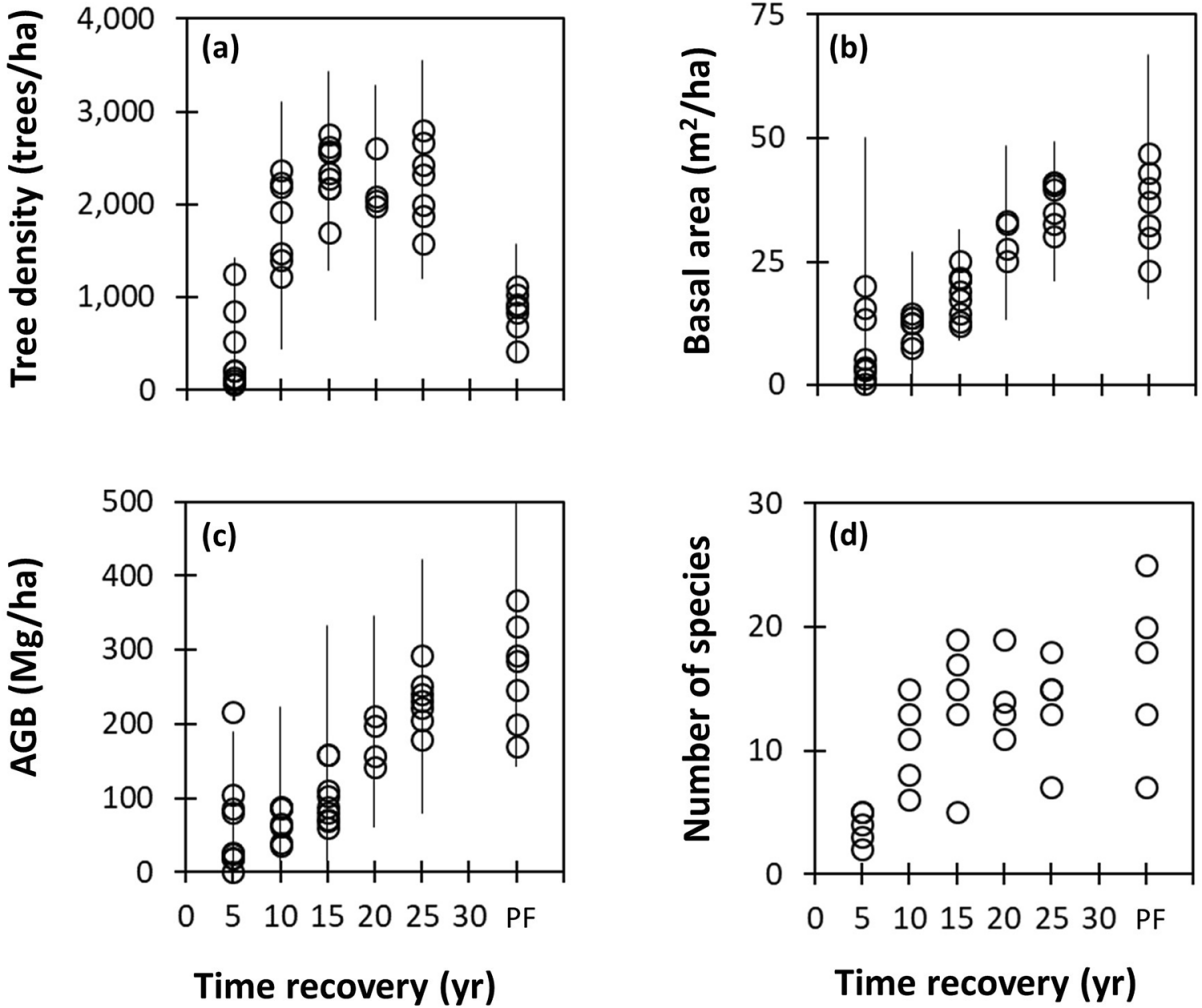
Forest structure recovery post-harvest, compared with intact protected mangrove forests, using measurement plots for (**a**) tree density, (**b**) basal area, (**c**) aboveground biomass (AGB), and (**d**) number of species. PF stands for Protected Forest, representing pristine mangrove conditions. Circles indicate mean value of forest structure from each plot, except for panel d which indicates total number of species. Error bars indicate standard deviation of overall mean.

Along carbon stocks, forest structure and species diversity also demonstrated recovery (Fig. 2, Table S1). Seedling densities were significantly higher in 5 year-old mangrove plots than in plots at any other stage (F_(5,13)_ = 28.321, *p* < 0.001). There were no pairwise differences when comparisons were made among other stages of regeneration post-harvest. The younger stands also had a significant number of large trees with an average diameter of 15.9 cm, larger than those found in stands 25 years post-harvest (12.2 cm). This is due to the presence of the seed trees in the harvest area (Fig. 2a). Seedling densities were highest in the younger stands, which suggests that seed trees and the surrounding greenbelt function well in providing propagules. However, it was observed that individual seed trees seem to die within 10 years after harvesting^4^. This does not appear to significantly disturb the regeneration process, the dieback likely to be due to lack of structural support and extreme winds. A study to compare the contribution of seed tree-derived propagules versus that of greenbelt-derived propagules, would help further understand regeneration dynamics. A similar situation of high propagule production was observed in the Hawaiian archipelago, with *Rhizophora mangle* producing propagules at a rate of 11 ton ha^−1^ yr^−1^, and becoming invasive^16^.

Mean tree diameters significantly differed across stand ages; the largest mean diameter at breast height (DBH) seen in protected mangroves (17.30 ± 12.00 cm), followed by 5-year-old stands (15.9 ± 0.4 cm), 25-year-old stands (12.15 ± 6.11 cm), 15-year-old stands (9.9 ± 0.5 cm), and 10-year-old stands (7.90 ± 4.71 cm). The fact that 5-year-old stands had a larger mean DBH is due to higher numbers of seed trees with larger diameters, compared to older stands.

The 25-year-old stands had the largest basal areas (BA) (37.12 ± 9.21 m^2^ ha^−1^), followed by protected mangrove, 15-year-old and 5-year-old stands. The 10-year-old stand had the lowest BA (10.58 ± 7.93 m^2^ ha^−1^), even lower than that of the 5-year-old stand (16.8 ± 3.3 m^2^ ha^−1^). These differences indicate that growth in the protected forests has been levelling off or even declining after tree maturity is reached, following a cycle of senescence and regrowth (Fig. 2b). In recently logged forests, the landscape was dominated by seedlings, yet fewer were naturally recruited in later years. Existing seedlings showed relatively low survival rates (around 25%) after 10 years. Overall, the mean BA significantly differed across stand ages.

*Rhizophora apiculata* dominated logged-over mangroves across different stand ages, with relative frequencies over 70%, but tended to decrease with increasing stand age. The species abundance gradually increased over time (Fig. 2d). *R. apiculata* reached its lowest frequency (32.7%) in protected forest, close to *Ceriops decandra* (27.2%). Likewise, *Bruguiera parviflora* and *B. gymnorrhiza* were more frequently found in older forest plots, as the established forest structure creates more favorable conditions for these genera to thrive. Species diversity in Bintuni Bay is highly dependent on the stand’s age and tidal zone, some species being present in the fringe mangrove (e.g., *Avicennia marina*) and others in the interior mangrove (e.g., *Ceriops tagal*).

Based on time-series Landsat imagery, an estimated 8,361 ha of mangrove area were harvested during 2001–2018, at an average of 465 ha harvested every year. Annual harvesting trends are presented in Fig. S2. This average (465 ha yr^−1^) is lower than the annual cutting projection for 2010–2020, of 800 ha yr^−1^ (ref.^17^), indicating that selective logging practiced in Bintuni Bay took just half the allowable cut, with extracted timber amounting to 237 m^3^ ha^−1^ (see Table S2). Such low logging intensity has significant potential to conserve biodiversity.

**Figure 3:**
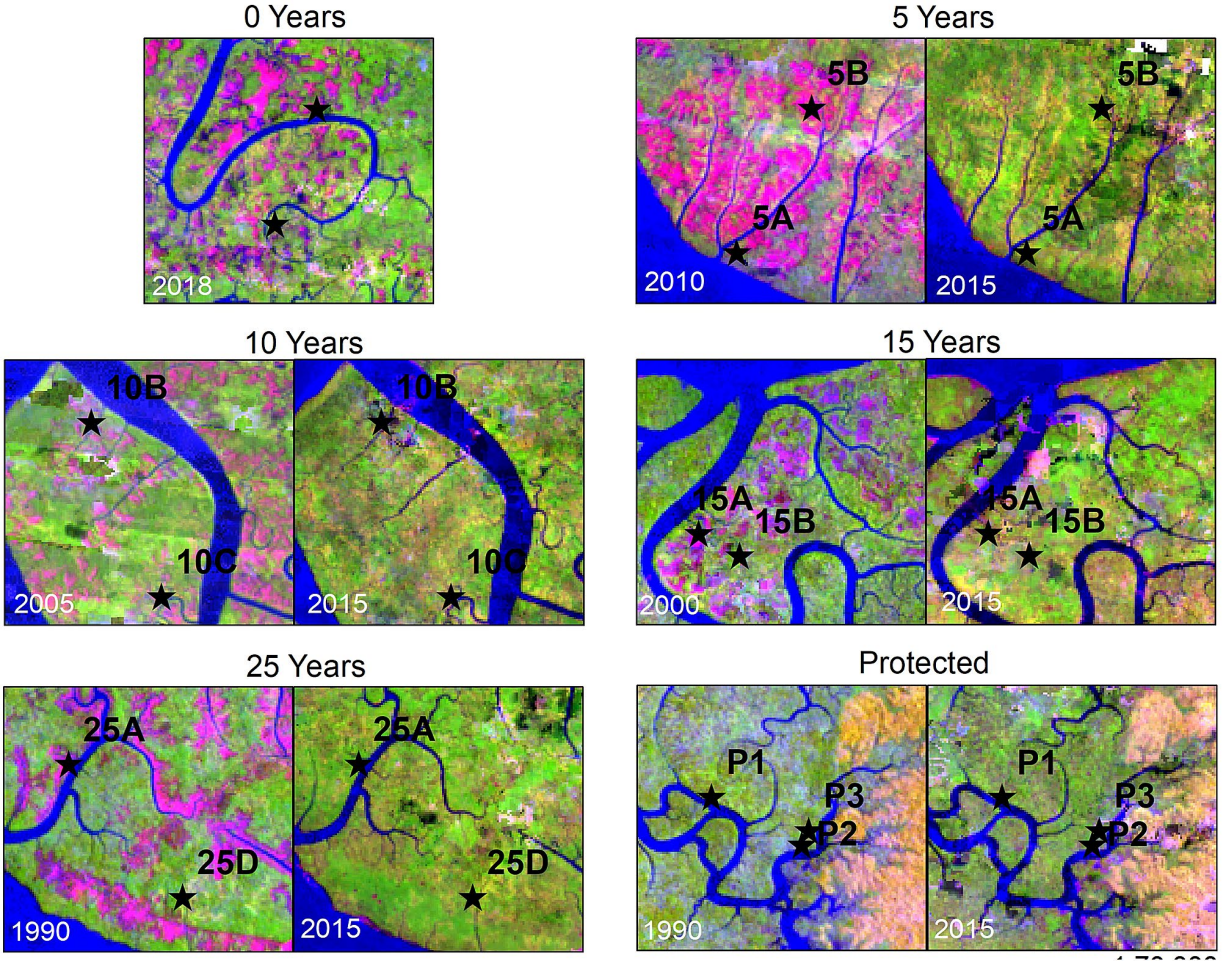
Landsat snapshots (1:70,000 scale) displayed in false colors (Red: Short-wave infra-red; Green: near infra-red; Blue: Red) show the recovery process after logging. Cloud-like pink patches in images of the initial years of logging reveal the ‘keyhole’ pattern of logging in this mangrove forest, from where measurements of carbon were taken. Comparisons between the year when logging took place (left panel of each year) and the situation in 2015 (right panel of each year) across all sampling plots, except for the Year 0 plot (sampled in 2018), show a regreening of logged areas.

Using the same Landsat images, rapid recovery of canopy cover was also demonstrated qualitatively. As shown in Fig. 3, all stands show regreening, including after 5 years post-logging. This indicates that improvement of vegetation cover secures plots from possible erosion, hence, depletion of soil carbon stocks. This impact could be particularly significant for estuarine hydro-geomorphic settings or the fringe mangroves found in the 15-year-old stands.

### Reduced impact logging and carbon stocks

**Figure 4:**
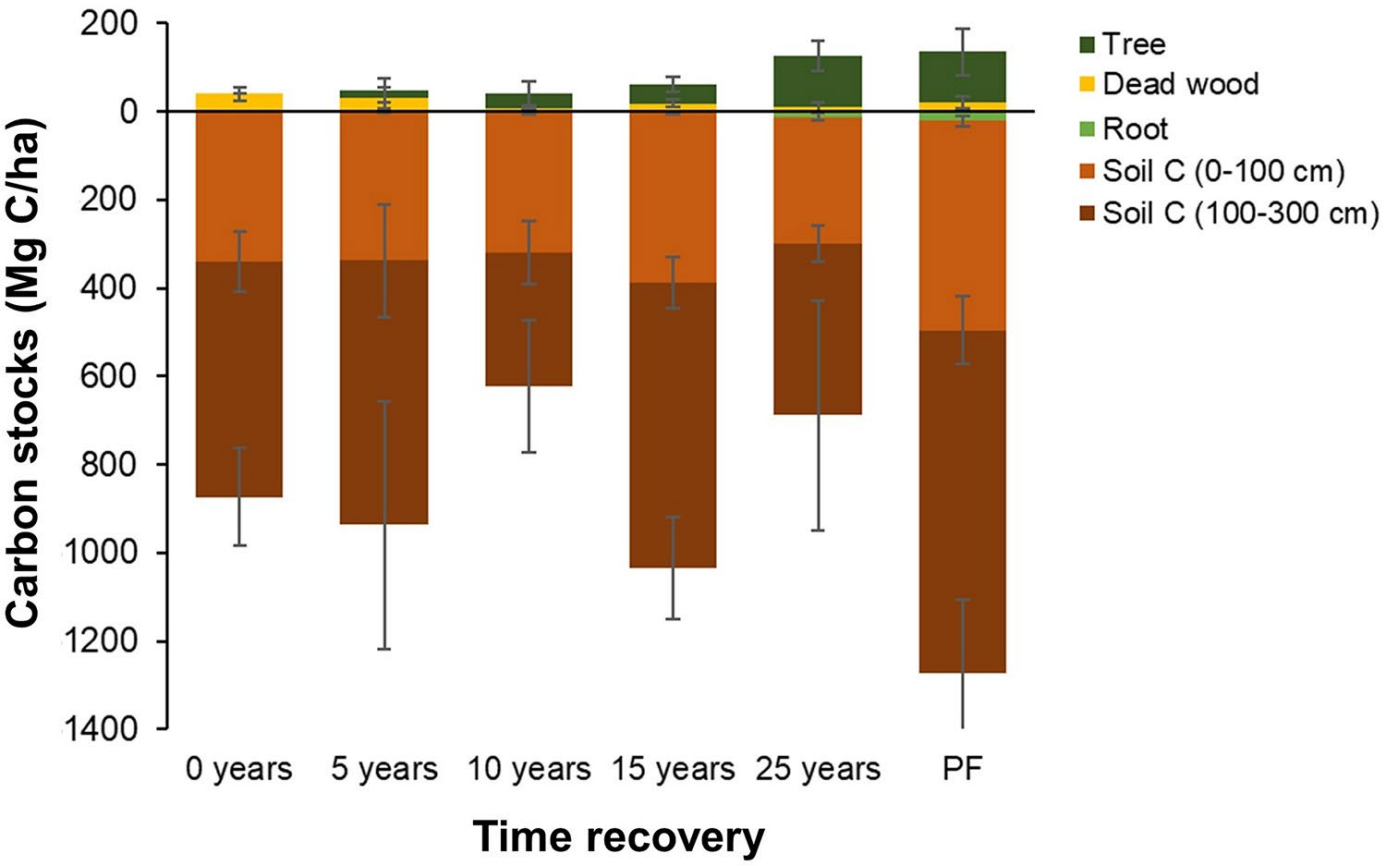
Total ecosystem carbon stock (TECS) variation across different ages of stands, representing the number of years of recovery, and protected forest (PF). Error bars indicate standard deviation of mean of respective carbon stock pool. Detailed sample size and site level carbon stocks data are provided in Table S3.

Within the Bintuni mangroves undergoing selective logging, we demonstrate that 25 years post-harvest, biomass carbon stocks are close to that of intact or protected mangroves. Secondary forest stands show a recovery in aboveground biomass carbon pools, from 5-year-old stands (16.43 ± 27.16) Mg C ha^−1^ to 25-year-old stands (114.52 ± 33.99) Mg C ha^−1^ (Fig. 4, Table S3).

Biomass carbon of 25-year-old manually logged forest stands are similar to that of intact protected undisturbed ones (*p* = 0.814). Although recovery was gradual, it can be observed as an independent and unbiased measure of sustainably managed mangrove forests. The recovery of canopy cover across differently aged stands is also a measurable indicator that rotational and selective logging secures aboveground biomass gains.

The annual mean of extracted biomass between 2015 and 2018 was 190 Mg ha^−1^ or approximately 95 Mg C ha^−1^ (Table S2), which is around 83% of fully recovered biomass carbon (Fig. 4). This amount is equivalent to 192 Mg (dry matter) ha^−1^, which is the same proxy for emission factors from aboveground biomass removal, to be used in assessing GHG emissions from managed mangroves when using IPCC’s Stock-Difference approach^11^. It was also observed that dead wood carbon stocks decreased as the stands got older. This suggests that logging residues may have decomposed or shifted elsewhere. The amount of dead wood carbon was relatively large compared with carbon sequestered in the biomass, 39.73 ± 14.54 Mg C ha^−1^ soon after logging and 10.76 ± 9.17 Mg C ha^−1^, 25 years after logging. This means that wood harvest waste was at least 40%.

Top 100 cm soil carbon stocks did not differ significantly across post-harvest stands, only varying between 354 ± 71 Mg C ha^−1^ in 10-year-old stands and 442 ± 57 Mg C ha^−1^ in 15-year-old stands. Even in newly logged sites, top 100 cm soil carbon stocks were relatively high at 381 ± 68 Mg C ha^−1^. Here, surface sediments were not significantly disturbed, and skidding trails were properly used for transporting the timber using local system called “Ongkak”. Skidding of logs on the wooden trails using “Ongkak” and loading them on the barges from the log yard at the end of “keyhole” shape logging plot has reduced soil surface disturbances. This suggests that reduced-impact logging preserves soil carbon stocks throughout the cutting cycle.

Deeper soil carbon stocks (i.e., at a depth of 100–300 cm) varied across all sites (Fig. 4, Table S3), confirming variation in carbon density reductions in deeper layers across all sites. This variation corresponds to different degrees of hydro-dynamic processes, under varied spatial and vertical geomorphology gradients^8,14^. Looking at a standardized depth of 300 cm to give a full soil carbon pool profile, TECS measurements were significantly different across all sites (*p* < 0.001, Fig. 4, Table S3); the lowest stocks (665 ± 201 Mg C ha^−1^) located in 10-year-old stands, and the largest stocks (1389 ± 199 Mg C ha^−1^) found in protected forests. The fact that post-harvest forests had lower TECS measurements compared to protected forests, was mainly attributed to lower soil carbon density, driven by hydro-geomorphology variation across sampling sites^8^. In addition, wood harvesting practice by coppicing the trees and not followed by excavating soil like in ponds development allows the conservation of soil carbon in generally estuarine landscape. Similar situation was found across Cambodian degraded and deforested mangroves when compared with 25 years old protected stands^18^. For example, examining the same complete profiles, we observed larger carbon densities in protected forests than in harvested sites (Table S3). Our findings are consistent with the 2013 IPCC Wetlands supplement^11^, suggesting that forest management in mangroves substantially affects biomass carbon pool, while soil pool remained unchanged.

The use of top-meter soil carbon stock pools as a standard soil profile from which to calculate coastal wetlands’ carbon storage, and emissions related to LULCC, is widely applied^19^ and suggested by the 2013 IPCC Wetlands Supplement for national-scale GHG reporting^11^. While we acknowledge the substantial roles that hydro-geomorphic variations play in controlling soil carbon stocks, a direct adoption of the Tier 1 global average, without incorporating hydro-geomorphic information, may lead to significant uncertainty. In Bintuni’s protected mangroves, we observed the mean of top-meter soil carbon stocks to be 474 Mg C ha^−1^, close to the IPCC Tier 1 value of 471 Mg C ha^−1^ (ref.^11^). Deeper soil sampling, however, revealed additional stocks of 776 Mg C ha^−1^ located at a soil depth of 100–300 cm, increasing total soil carbon stock to 1250 Mg C ha^−1^. This implies that soil sampling below 100 cm is necessary in mangroves, and particularly useful to calculate carbon losses from land conversion that excavates soil to depths of over 100 cm (e.g., aquaculture pond development). Overall, TECS measurements in Bintuni Bay’s protected mangroves are 60% higher than the global average (856 ± 32 Mg C ha^−1^)^10^. Such high TECS measurements should attract the development of project-based activities for climate change mitigation.

### Continued sediment accretion and carbon burial

There is a consistent increase pattern of historical and contemporary cumulative sediment accretion across hydro-geomorphic settings and stand ages (Fig. 5 and Table 1). Nevertheless, the rates of contemporary sediment accretion are higher compared to the historical ones, except in fringe protected mangrove site (Table 1). Historical sediment reconstruction results in net sediment accumulation, due to both biological (e.g., litterfall, benthic algae mat growth, root production and decomposition) and physical (e.g., erosion, compaction and groundwater shrink/swell) processes over the course of a decade^20^. Under shorter observation period, high contemporary accretion in the surface layer suggest a larger net sediment accumulation with less sediment losses from decomposition, erosion, and compaction.

**﻿Figure 5:**
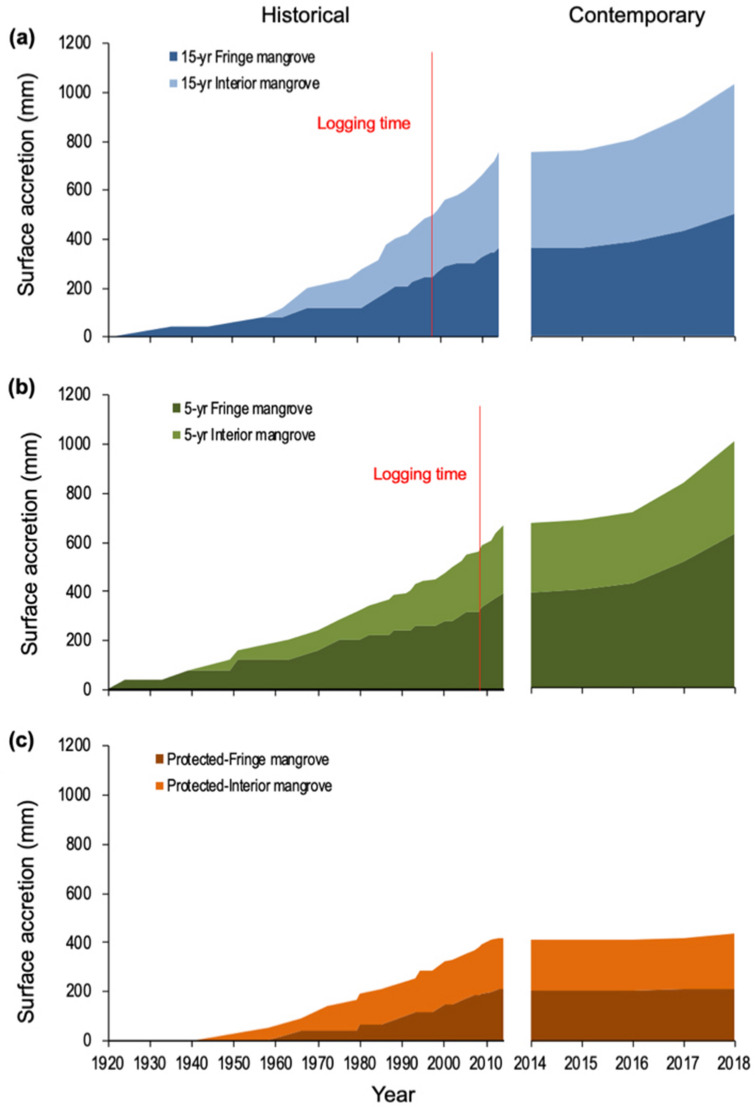
Historical cumulative vertical accretion derived from ^210^Pb radionuclide technique (left-hand panels) and contemporary vertical accretion from marker horizons (right-hand panels), measured in different hydro-geomorphic settings and stand ages: (**a**) 15-year-old stands; (**b**) 5-year-old stands; and (**c**) protected mangroves.

**Table 1:** Comparison of contemporary (MH derived) and historical (^210^Pb derived) accretion rates and carbon accumulation rates in 5-year-old, 15-year-old and protected stands, in fringe and interior hydro-geomorphic settings. Accretion and carbon accumulation rates are presented as mean ± SD.

Site	MH observation period (year)	MH derived accretion rate (mm yr^−1^)	MH derived C accumulation (Mg ha^−1^ yr^−1^)	^210^Pb sediment age (year)	^210^Pb derived accretion rate (mm yr^−1^)	^210^Pb derived C accumulation (Mg ha^−1^ yr^−1^)
*Fringe—*15 years post-harvest	4.2	9 ± 11	3.51 ± 41.1	92	6.45 ± 3.5	3.65 ± 1.95
*Interior—*15 years post-harvest	4.2	9 ± 6	1.00 ± 0.67	69	8.27 ± 4.49	2.67 ± 0.85
*Fringe—*5 years post-harvest	4.2	16 ± 11	13.08 ± 8.97	107	5.84 ± 3.83	1.13 ± 0.78
*Interior—*5 years post-harvest	4.2	8 ± 7	2.90 ± 2.31	79	9.87 ± 19.39	3.39 ± 7.24
*Fringe—*protected area	3.0	0.5 ± 0.1	0.07 ± 0.02	74	3.9 ± 0.7	0.50 ± 0.20
*Interior—*protected area	3.0	3 ± 3	0.91 ± 0.97	87	2.5 ± 0.3	0.90 ± 0.40

*MH* marker horizon.

Direct measurements of contemporary sediment vertical accretion show that the younger the stands, the more sediment was accreted vertically (Fig. 5b). This may be associated with the large amount of deadwood materials left on the surface after logging event (see Table S3). These fresh woody debris could potentially trap more sediments and enhance accretion before they are decomposed and contribute to accretion as well. While bioturbation has been known as one of main limitations in coastal sedimentation studies, particularly those who used radionuclides sediment dating approach^21–23^, here accretion may be additionally affected by logging event. We underline that such logging event may have caused surface sediment dynamics at substantial degree despite manual logging approach was implemented.﻿

Cumulative sediment measured over 4.2 years reached up to 62 mm in 5-year-old stands, 38 mm in 15-year-old stands, and 8 mm in protected mangroves. Accretion was also greater in fringing mangroves than in interior mangroves, consistent with the historical sediment accretion rates. This indicates that the majority of suspended sediment from incoming high tides is initially deposited in fringing areas, where roots and trunks slow down the current’s velocity, and thus the energy required to settle sediments.

Table 1 shows that historical vertical accretion rates derived using the ^210^Pb radionuclide technique ranged between 3 and 13 mm yr^−1^, while contemporary vertical accretion rates derived from marker horizons (MH), ranged between 1 and 16 mm yr^−1^. These rates were higher than the rate of global sea level rise, which was 3.2 (2.8–3.6) mm yr^−1^ between 1993 and 2010^24^. However, fringe mangroves are also reportedly prone to erosion^20^. This implies that the hydro-geomorphic setting should be considered when conserving pristine mangroves or restoring degraded mangroves in areas subject to forestry practices, so as to support adaptation to climate-driven sea-level rise.

Based on carbon density estimates, using the soil cores, carbon burial rates were 0.1–13 Mg ha^−1^ yr^−1^ for MH, similar to the ^210^Pb radionuclide technique results of 1–3 Mg ha^−1^ yr^−1^. This historical carbon burial rates are similar to that found in estuarine mangroves in Vietnam and Palau^25^, and within the range of global numbers previously reported, 1.3–2.0 Mg ha^−1^ yr^−1^ (ref.^26^). The wide range of sediment age in Table 1 suggests that even when logging operations have been underway for over 25 years, soil carbon remains secure, including in the most recently logged plots. If the ecosystem is well managed and disturbance is minimized, soil carbon can be stored for long periods. In protected mangroves, soil carbon is key to secure permanence.

Leaving seed trees and greenbelts supported rapid canopy recovery and sedimentation. The observed sediment accretion rates by MH suggests that there is a lot of sediment entering these systems supporting a greater net sediment accumulation, while the ^210^Pb radioactive tracer suggests that this dynamic has been occurring for the last 100 years and results in a lower net sediment accumulation. Through efforts to refine and indicate the constraints of global estimates of organic carbon burial rates, it has been confirmed that site-specific measurements are key to reduce uncertainties^21,27^. However, measurements may be extended to include mudflat hydrogeomorphic settings and identify organic matter sources, to capture the full fate of sedimentary carbon.

## Conclusions

The keyhole harvest pattern used during selective logging of mangrove allowed the canopy to recover relatively quickly. In addition to the low impact of this harvest method, the availability of propagules from seed trees and greenbelt allowed natural regeneration to take place. As a result, biomass carbon was fully restored after one cutting cycle, that has recently been expanded from 25 to 30 years. Although a few species dominated in the recovering stands, in later years species diversity was improved and soil carbon was maintained.

It is demonstrated that soil carbon stocks in selectively logged mangroves were preserved over time. Reduced disturbance, due to the low logging intensity, prevented soil carbon stocks from being released and depleted. Stock-Difference emission factors for GHG inventories can now be developed to include soil carbon stock change despite spatially hydrogeomorphic driven variation need to be considered. Permanence may also be evaluated, in the context of incorporating mangrove forests into climate change mitigation strategies and thus mangroves may be included in the REDD + mechanism.

Sedimentation in coastal forest ecosystems, including mangroves, has a significant role to play in maintaining and recovering TECS and vertical sediment accretion. The soil carbon sequestration process, consistently proven using both historical and contemporary approaches, suggests that mangrove forest soils provide active sinks when managed sustainably. Top-meter assessment of soil carbon stocks significantly underestimate mangrove ecosystems, including those in Bintuni Bay. In most estuarine mangroves, soil carbon stocks can be double when deeper samples are included. Although no disturbance is expected in such a remote area, extending the standard to a depth more than 1 m would encourage the participation of land managers and the private sector in climate change mitigation and adaptation actions.

## Methods

### Study site

This study took place in managed mangrove forests in the southern part of Bintuni Bay, West Papua Province, Indonesia (Fig. S1). In Bintuni Bay, selective, rotational logging of mangroves has occurred since 1989^4,5^, permitting a study of changes in TECS from this type of management. The study was carried out in an 82,120 ha mangrove forest concession, operated by a private forestry company that harvests mangrove for woodchip and follows a 30-year harvest rotation period. Mangroves in Bintuni Bay grow within an estuarine hydro-geomorphic setting and are characterized by tall stands dominated by *Rhizophora* spp. and deep organic soils^8^.

### Datasets

We collected primary field data and complimented them with previously-collected data from the same study site. Primary field data assessments were carried out at sites 5 and 15 years post-harvest, looking into forest structure, carbon stocks, contemporary and historical carbon burial. Figure S2 details the size and layout of plots assessed in primary field data collection. Ancillary datasets on forest structure and TECS for undisturbed forest and forest plots 0, 10 and 25 years post-harvest, were obtained from refs. 4 and 8. Collection methods for these datasets are described in detail in the cited studies. Historical data on carbon burial in protected forest sites were obtained from ref.^21^. Cloud-free time-series Landsat composite images were used to calculate the area of forest loss inside the logging concession each year from 2001 until 2018.

### Carbon stocks analysis

We completed the carbon stock assessment in areas harvested in a keyhole shape, randomly selecting 3–5 plots of mangrove stand logged 0, 5, 10, 15 and 25 years ago, as well as in the protected area, to assess the effect of logging rotation on TECS. Plot and sub-plot design and layout for primary data collection are shown in Fig. S3a,b. We assessed above and belowground biomass, woody debris and soil carbon pools, following globally-applied protocols for mangrove TECS assessment^28^. To capture post-logging effects within the keyhole-shaped harvest area, we modified plot layout by reducing the number of sub-plots from six to four for mangrove sites 5 and 15 years post-harvest (see Fig. S3b). In these locations, carbon stocks are summarized into a single mean value, due to use of a large circular plot design to cover the presence of big trees (DBH > 50 cm). Species-specific allometric equations to convert diameter at breast height into biomass were used and are summarized in Table S4. We calculated the relative frequency of species based on the number of trees and seedlings encountered for each species, relative to the total number of trees in the surveyed area. We followed common procedures for mangrove forest surveying to calculate basal area (m^2^ ha^−1^) and tree density (tree ha^−1^)^29^. Dead wood carbon stocks were assessed by using planar intercept technique at each sub-plot^28^. We collected soil samples for carbon stocks presented in Fig. 4 from six different depth intervals, namely 0–15, 15–30, 30–50, 50–100, 100–200, 200–300 cm. Following a standard soil sample collection for coastal wetlands^30^, approximately 5 cm soil thickness was collected from the mid-point of these six intervals. Total soil carbon stocks at each sub-plot are the summary of carbon stocks from all six depth intervals—fixed depth approach.

### Sediment accretion and carbon burial

Sediment accretion rate was calculated by using two approaches, namely ^210^Pb radionuclide sediment dating and Marker-Horizon (MH), which respectively represent historical and contemporary sediment accretion temporal scales. For ^210^Pb activity analysis, two sediment cores were collected for each stand representing 5 and 15 years of forest regeneration. We used the standardized sediment collection technique^25^ and then dated the cores with ^210^Pb using the Constant Rate of Supply (CRS) model, described in ref.^31^. The CRS method is used in systems such as mangroves because sediment accumulation rates can vary while the supply rate of ^210^Pb has been relatively constant^23^. Briefly, sediment cores were collected to a depth of 50 cm and sectioned into 2 cm intervals for the first 20 cm and 4 cm intervals for the remaining core depth. Intervals were then analyzed for ^210^Pb activity as described in ref.^25^. Total activities were then plotted against cumulative mass and fit with a regression line using radioactive decay law (Fig. S4)^25^. We used the asymptote to identify intervals that approached the value of that asymptote and then averaged those interval values to estimate supported ^210^Pb values. We attributed values that were greater or less than the radioactive decay line, which was most apparent in 5 years post-harvest forests (Table S5), to mixing, bioturbation, or detector error. Sediment mixing is one of the main limitations for sediment dating approach using particularly radionuclide tracer and it is commonly due to bioturbation from macrobenthos, root productivity and mortality, and seasonally driven erosion. These processes were particularly observed for mudflat habitat near protected forests where sediment accumulation is highly driven by seasonal hydrodynamics^21^ and resulted in cores that could not be dated.

Contemporary carbon burial was observed in forest sites 5 and 15 years post-logging, as well as in an protected forest site, using the Marker-Horizon (MH) approach combined with carbon density^32^. In 2014, several replicates of MH were installed in sites at 5 and 15 years post-harvest, and in protected forest. We subsequently observed accreted sediments every 6–8 months between 2014 and 2018. The contemporary sediment accretion rate was calculated by dividing the vertical thickness of accreted sediments over the measurement period, at each sampling location. Finally, the sediment carbon accumulation rate (Mg C ha yr^–1^) was estimated by multiplying the accretion rate (cm yr^–1^) by the carbon density (g C cm^–3^). While bulk density data could be established via soil samples (Table S5), carbon content was established from the mean value of soil sampled for carbon stocks. Consequently, some soil accretion layers have similar carbon content (i.e., 0–15 cm, 15–30 cm, 30–50 cm, see Table S5).

### Satellite-based canopy cover change analysis

We estimated the area of forest harvested inside the logging concession annually between 2001 and 2018 by re-analyzing the Tree Loss dataset (v.16) developed at University of Maryland with LANDSAT time-series imagery^33^. This dataset measures the removal of trees (over 5 m high) if the canopy cover of a 30 m × 30 m land unit (1 LANDSAT pixel) falls below 30%. Our re-analysis included harvested areas that were missed in the original dataset. We did this by scanning a sequence of eighteen annual cloud-free LANDSAT composite images, developed in Google Earth Engine^34^, before applying supervised classification to this sequence of images to extract Tree loss^35^. We then determined losses in natural mangrove forest area by excluding Tree Loss pixels outside of the area occupied by mangrove forests in the year 2000, using a mangrove forest mask^35^. We also displayed multi-date Landsat digital images (scale 1:70,000) with an RGB (Red, Green and Blue channels) color model to show qualitatively that the recovery process after logging can be seen from space-borne cameras. The imagery makes areas of land covered with vegetation appear green, by placing the Shortwave infrared (SWIR) reflectance in the Red channel, the Near-infrared (NIR) reflectance in the Green channel, and the Red reflectance in the Blue channel. Logged areas look red/pink because vegetation has been cleared. The resulting loss of chlorophyll considerably reduces the reflection of the NIR (in the Green channel), while increased exposure to soil and wood debris increases the reflection of SWIR (in the Red channel). When vegetation grows, the opposite happens: the reflection of the NIR increases (in the Green channel) because of chlorophyll, while the reflection of SWIR decreases (in the Red channel).

### Statistical analysis

We compared forest structure and carbon stocks across study sites by using analysis of variance (ANOVA) with a Bonferroni multiple comparison test. We applied a normality test prior to ANOVA and employed a non-parametric Kruskal–Wallis test if the dataset was not normally distributed. All statistical analyses were performed using R statistic.

## Supplementary Information

Supplementary Information.
