# Supplementary material for: Mangrove selective logging sustains biomass carbon recovery, soil carbon, and sediment
Source: Sci Rep. 2021 Jun 10;11:12325. doi: 10.1038/s41598-021-91502-x (PMC8192934; doi:10.1038/s41598-021-91502-x)
Supplement: Supplementary file 1 — Supplementary Information. [file 41598_2021_91502_MOESM1_ESM.docx]

***Supplementary Information***

**Mangrove selective logging sustains biomass carbon recovery, soil carbon, and sediment**

Daniel Murdiyarso ^1,2^, Sigit D Sasmito ^1,3^, Mériadec Sillanpää ^4,5^, Richard MacKenzie ^6^, and David Gaveau ^1,7^

1. Center for International Forestry Research, Jl. CIFOR, Situgede, Bogor 16115, Indonesia
2. Department of Geophysics and Meteorology, IPB University, Bogor 16680, Indonesia
3. NUS Environmental Research Institute, National University of Singapore, 21 Lower Kent Ridge Road, Singapore, 119077, Singapore
4. Green Forest Product & Tech. Pte. Ltd., 3 Shenton Way, Singapore 068805, Singapore
5. Department of Geography, National University of Singapore, 1 Arts Link, Singapore 117570, Singapore
6. USDA Forest Service, Pacific Southwest Research Center, Institute of Pacific Islands Forestry, 60 Nowelo St., Hilo, HI 96720, USA

7. TheTreeMap, Bagadou Bas 46600 Martel, France


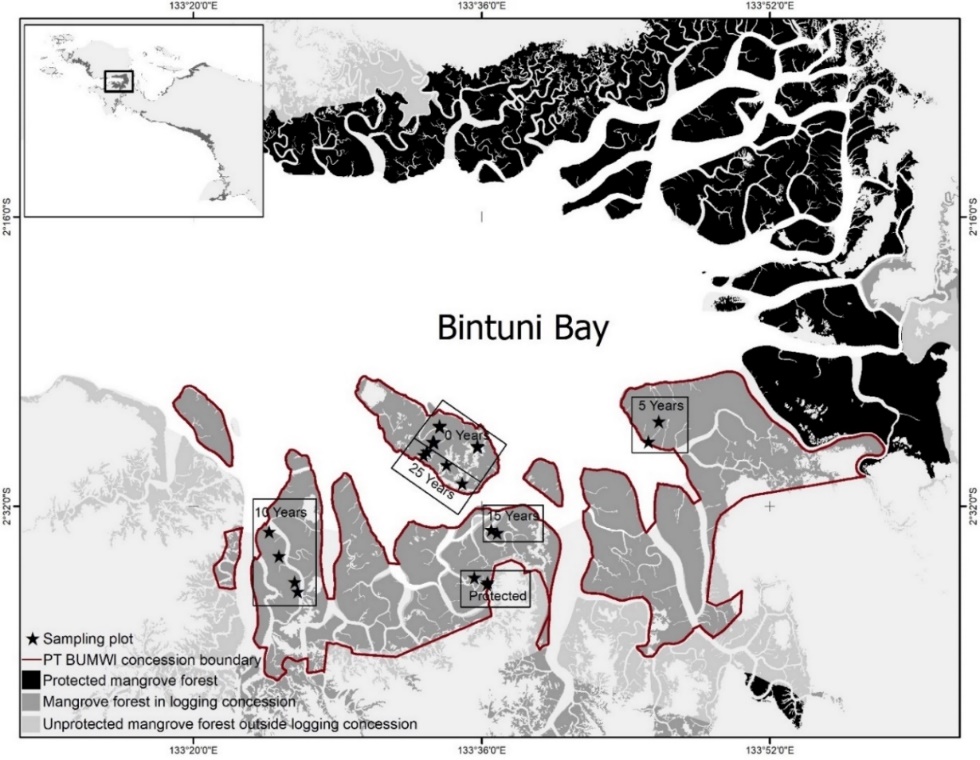


**Figure S1.** Sampling sites scattered across an 82,120-ha logging concession in southern Bintuni Bay, West Papua. Plots represent stands 0, 5, 10, 15, and 25 years post-harvest, and protected forest within the concession area (Source: Gaveau et al. 2019 and Ministry of Forestry, Republic of Indonesia 2013).


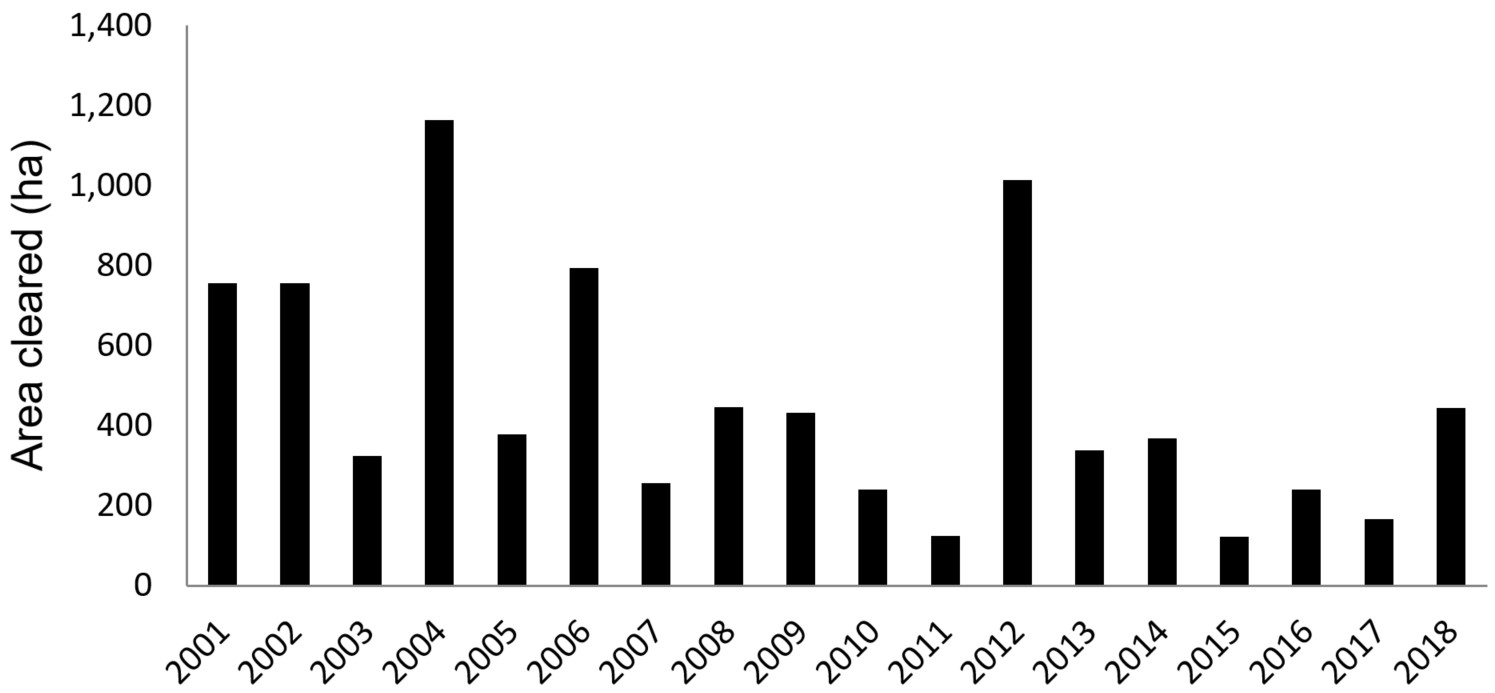


**Figure S2.** Estimated mangrove forest area harvested annually from 2001 to 2018 under the ‘keyhole’ silviculture practice in the Bintuni concession. Approximately 8,361 ha of the 82, 120 ha mangrove concession have been harvested during this period. This estimation follows one calendar year and based on a re-analysis of the *Tree Loss* dataset (v.16) developed at University of Maryland with LANDSAT time-series imagery^[[1]](#endnote-1)^. Estimated harvest area may be subject to error, due to road construction or urban development occurring in the peripheries of the mangrove forest.

| 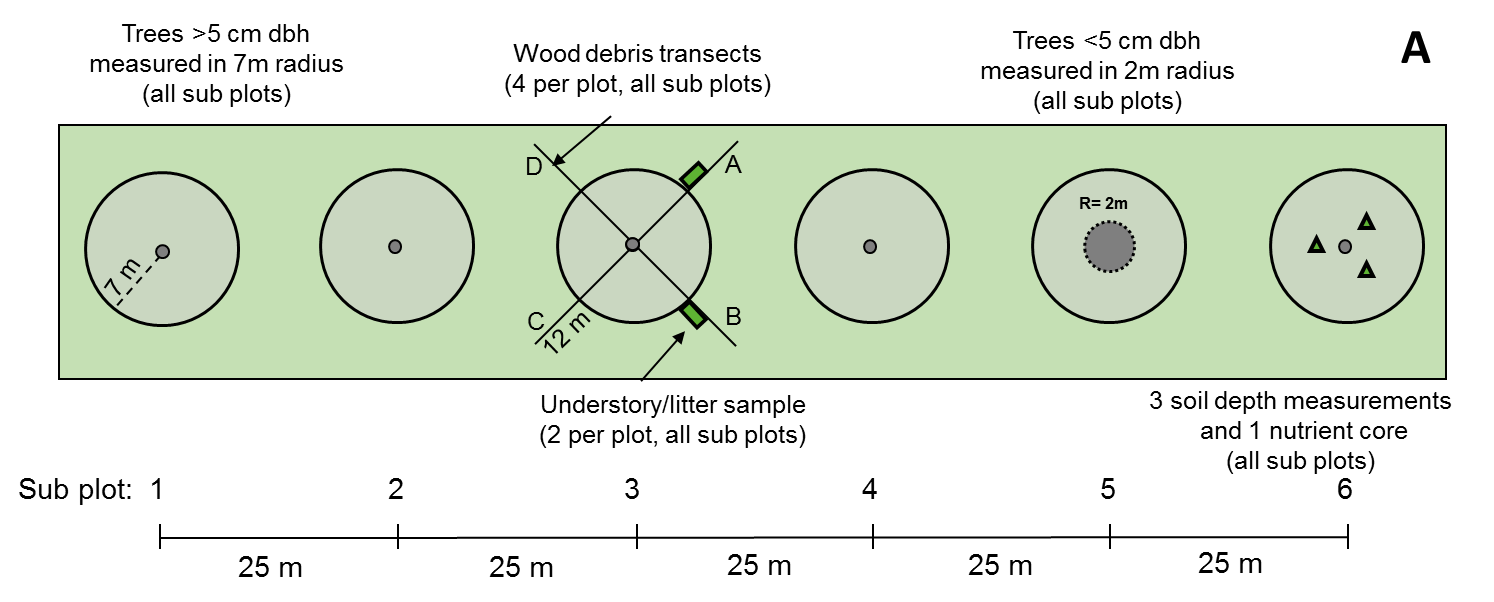 |
| --- |
|  |
| 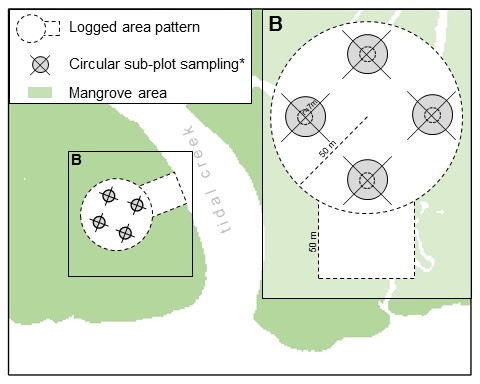 |

**Figure S3.** Plot design and layout used in carbon stock assessments in (A) an elongated plot with six sub-plots for 25-year-old stands and protected mangroves, and (B) a keyhole shape plot with four sub-plots for 5 and 15-year-old stands. A 50 m diameter was used to cover the presence of big trees (DBH>50 cm); carbon stocks were therefore summarized into a single mean for each site.

**Figure S4**. ^210^Pb activity against cumulative mass. The shaded areas indicate the supported ^210^Pb activity derived from the Constant Rate of Supply (CRS) approach, when total ^210^Pb activity approaches the asymptote of the line fit to the data using the radioactive decay formula.

**Table S1.** Forest structure characteristics in each age class of logged-over mangrove forest, compared with protected or unlogged mangrove forests (additional data for 5, 15 and 20-year-old forest stands are from the Sillanpää et al. 2017 databases)

| **Stand age/ status** | **Plot** | **Stand density (individual ha^-1^)** | | **Basal area**  **(m^2^ ha^-1^)** | **DBH**  **(cm)** | **Relative frequency (%) of species** |
| --- | --- | --- | --- | --- | --- | --- |
|  |  | **Seedling (DBH<5 cm)** | **Tree (DBH>5 cm)** |  |  |  |
| 5 years | 5A | 38,217 ± 8,369 | 1,251 ± 173 | 20.0 ± 3.8 | 15.6 ± 1.8 | Ra (41.5), Bp (53.7), Bg (2.4), Sa (2.4) |
|  | 5B | 55,932 ± 2,588 | 845 ± 287 | 13.5 ± 4.3 | 16.3 ± 4.1 |  |
|  | 5C | 39,591 ± 19,777 | 520 ± 283 | 15.7 ± 34. | 16.6 ± 13.9 |  |
|  | 5D | 3,780 ± 2,191 | 130 ± na | 1.5 ± 4.0 | 28.4 ± na |  |
|  | 5E | 8,356 ± 8,641 | 217 ± 99 | 5.5 ± 8.0 | 22.6 ± 11.9 |  |
|  | 5F | 6,526 ± 3,252 | 65 ± na | 0.3 ± 0.2 | 7.5 ± 2.6 |  |
|  | **Mean** | 25,400 ± 21,964 **a** | 505 ± 467 **a** | 9.4 ± 8.1 **a** | 17.8 ± 7.1 **a** |  |
| 10 years | 10A | 19,708 ± 14,170 | 1,224 ± 772 | 7.50 ± 5.58 | 8.06 ± 3.61 | Ra (79.6), Bp (15.7),  Bg (2.7), Ct (0.9),  Rm (0.7), Sa (0.2) |
|  | 10B | 12,854 ± 9,328 | 1,928 ± 1,181 | 12.52 ± 10.80 | 7.28 ± 5.46 |  |
|  | 10C | 8,795 ± 4,866 | 1,820 ± 1,062 | 13.57 ± 7.06 | 8.67 ± 4.46 |  |
|  | 10D | 8,044 ± 1,554 | 1,398 ± 952 | 8.73 ± 7.80 | 7.62 ± 4.65 |  |
|  | **Mean** | 12,350 ± 9,516 **a** | 1,593 ± 981 **bce** | 10.58 ± 7.93 **a** | 7.90 ± 4.71 **b** |  |
| 15 years | 15A | 6,568 ± 1,047 | 2,567 ± 210 | 25.2 ± 6.1 | 10.4 ± 0.9 | Ra (72.1), Bp (11.9), Bg (0.2), Rm (13.9), Ct (1.6), Xg (0.2) |
|  | 15B | 8,758 ± 1,810 | 1,706 ± 409 | 12.1 ± 3.0 | 9.4 ± 0.8 |  |
|  | 15C | 2,586 ± 398 | 2,763 ± 304 | 21.8 ± 7.4 | 9.4 ± 1.3 |  |
|  | 15D | 5,173 ± 3,345 | 2,286 ± 976 | 21.5 ± 10.0 | 9.8 ± 2.8 |  |
|  | 15E | 4,510 ± 2,550 | 2,178 ± 217 | 12.8 ± 3.1 | 8.0 ± 0.8 |  |
|  | 15F | 4,775 ± 3,329 | 2,178 ± 383 | 14.4 ± 2.6 | 8.7 ± 1.1 |  |
|  | **Mean** | 5,395 ± 2,088 **a** | 2,279 ± 365 **cdf** | 18.0 ± 5.5 **a** | 9.3 ± 0.8 **b** |  |
| 20 years | 20A | 1,990 ± 1,572 | 2,611 ± 672 | 33.3 ± 7.5 | 11.4 ± 1.2 | Ra (85.8), Bp (8.2), Bg (0.5), Rm (4.4), Ct (0.7), Xg (0.4) |
|  | 20B | 3,342 ± 2,060 | 1,983 ± 1,226 | 25.0 ± 11.6 | 12.2 ± 3.1 |  |
|  | 20C | 3,051 ± 1,544 | 2,037 ± 208 | 27.5 ± 6.5 | 12.0 ± 1.2 |  |
|  | 20D | 5,889 ± 5,415 | 2,080 ± 1,031 | 32.7 ± 15.8 | 12.6 ± 3.8 |  |
|  | Mean | 3,568 ± 1,653 **a** | 2,178 ± 292 **ef** | 29.6 ± 4.0 **b** | 12.0 ± 0.5 **ab** |  |
| 25 years | 25A | 6,725 ± 7,101 | 2,806 ± 751 | 39.73 ± 5.58 | 12.48 ± 4.97 | Ra (80.3), Bp (12.7),  Rm (4.6), Bg (1.1),  Ct (1.1), Sa (0.2), Xg (0.1) |
|  | 25B | 1,365 ± 1,145 | 2,665 ± 607 | 41.08 ± 7.04 | 12.82 ± 5.65 |  |
|  | 25C | 4,613 ± 2,112 | 2,318 ± 1,118 | 32.68 ± 7.85 | 11.90 ± 6.18 |  |
|  | 25D | 6,313 ± 5,556 | 2,427 ± 805 | 34.99 ± 13.84 | 11.30 ± 7.50 |  |
|  | **Mean** | 4,754 ± 4,855 **a** | 2,554 ± 809 **def** | 37.12 ± 9.21 **b** | 12.15 ± 6.11 **ab** |  |
| Protected forest | KPPN1 | 8,505 ± 6728 | 899 ± 397 | 29.92 ± 7.27 | 16.75 ± 12.03 | Ra (32.7), Cd (27.2),  Bp (14.7), Bg (9.2), Rm (8.5), Xg (2.2), Xm (1.8) |
|  | KPPN2 | 8,640 ± 5948 | 1,116 ± 352 | 32.48 ± 15.08 | 16.34 ± 10.22 |  |
|  | KPPN3 | 9,006 ± 7189 | 932 ± 182 | 40.07 ± 16.28 | 18.98 ± 13.77 |  |
|  | **Mean** | 8,717 ± 6242 **a** | 982 ± 320 **b** | 34.16 ± 13.42 **b** | 17.30 ± 12.00 **a** |  |
|  |  | F_(5,21)_= 2.917,  *p*>0.05 | F_(5,21)_= 26.593, *p*<0.001 | F_(5,21)_= 20.076, *p*<0.001 | F_(5,21)_= 6.300, *p*<0.01 |  |

Am: *Avicennia marina*, Bg: *Bruguiera gymnorrhiza*, Bp: *Bruguiera parviflora*, Cs: *Camptostemon schultzii*, Cd: *Ceriops decandra*, Ct: *Ceriops tagal*, Ra: *Rhizophora apiculata*, Rm: *Rhizophora mucronata*, Sa: *Sonneratia alba*, Xg: *Xylocarpus granatum,* Xm*: Xylocarpus moluccensis*

**Table S2.** Harvest area and volume of timber extracted between 2015–2018 in Bintuni Bay, West Papua

| **Harvest year** | **Harvest area (ha)^[[2]](#endnote-2)^** | **Dry weight (tons)^[[3]](#endnote-3),^^[[4]](#endnote-4),^^[[5]](#endnote-5)^** | **Harvest rate (tons ha^-1^)** | **Harvest rate (m^3^ ha^-1^)*** |
| --- | --- | --- | --- | --- |
| 2015 | 220.73 | 40000.0 | 181.22 | 226.52 |
| 2016 | 254.49 | 52000.0 | 204.33 | 255.41 |
| 2017 | 246.61 | 47949.0 | 194.43 | 243.04 |
| 2018 | 302.08 | 54207.0 | 179.45 | 224.31 |
| Total | 1023.91 | 194156.0 | - | - |
| **Mean** | **255.98** | **48539.00** | **189.86** | **237.32** |

* Calculated from tons of exported wood (Bone-Dry Metric Ton, BDMT, written as ‘Dry Weight’ in the table), assuming a wood density of 0.8 g cm^-3^

| **Table S3.** Summary of carbon stocks (Mg C ha^-1^) in each pool across different post-logging ages. | | | | | |
| --- | --- | --- | --- | --- | --- |
| **Land use type** | **Sampling plot** | **Aboveground Carbon** | **Belowground Carbon** | **Deadwood Carbon** | **Soil Carbon – 100 cm** |
| 0 years | 0A | 0 ± 0 (6) | 0 ± 0 (6) | 33.37 ± 13.21 (6) | 310.29 ± 59.44 (6) |
| 0 years | 0B | 0 ± 0 (6) | 0 ± 0 (6) | 39.43 ± 17.55 (6) | 326.23 ± 53.89 (6) |
| 0 years | 0C | 0 ± 0 (6) | 0 ± 0 (6) | 46.38 ± 11.61 (6) | 386.52 ± 83.91 (6) |
| **0 years mean** |  | **0 ± 0 (18)a** | **0 ± 0 (18)a** | **39.73 ± 14.54 (18)a** | **341.01 ± 67.69 (18)a** |
| 5 years | 05-01 | 12.87 ± 21.83 (6) | 1.61 ± 3.19 (6) | 30.13 ± 15.5 (5) | 361.82 ± 63.96 (6) |
| 5 years | 05-02 | 9.36 ± 21.81 (6) | 1.49 ± 3.53 (6) | 19.17 ± 11.04 (5) | 370.48 ± 146.28 (6) |
| 5 years | 05-03 | 8.81 ± 11.26 (6) | 1.84 ± 2.9 (6) | 26.36 ± 16.74 (5) | 380.11 ± 147.95 (6) |
| 5 years | 5A | 102.02 ± na (1) | 6.43 ± na (1) | 104.98 ± 65.42 (4) | 107.86 ± 41.72 (4) |
| 5 years | 5B | 40.41 ± na (1) | 3.42 ± na (1) | 41.31 ± 25.41 (4) | 254.04 ± 77.07 (4) |
| **5 years mean** |  | **16.43 ± 27.16 (20)b** | **1.97 ± 3.08 (20)b** | **30.86 ± 23.67 (17)ab** | **336.29 ± 128.42 (20)a** |
| 10 years | 10-01 | 41.35 ± 44.09 (6) | 3.67 ± 4.02 (6) | 7.17 ± 4.33 (6) | 364.14 ± 81.51 (6) |
| 10 years | 10-02 | 30.78 ± 7.59 (6) | 3.57 ± 1.75 (6) | 9.27 ± 4.25 (6) | 276.39 ± 79.24 (6) |
| 10 years | 10-03 | 29.37 ± 15.04 (6) | 2.97 ± 1.69 (6) | 6.49 ± 1.98 (6) | 306.92 ± 39.44 (6) |
| **10 years mean** |  | **33.83 ± 26.18 (18)c** | **3.4 ± 2.57 (18)c** | **7.65 ± 3.67 (18)c** | **315.82 ± 71.47 (18)a** |
| 15 years | 15-01 | 37.63 ± 16.4 (6) | 3.7 ± 2.59 (6) | 18.41 ± 7.4 (6) | 417.8 ± 41.62 (6) |
| 15 years | 15-02 | 51.81 ± 21.2 (6) | 5.01 ± 2.29 (6) | 13.69 ± 8.41 (6) | 385.3 ± 30.52 (6) |
| 15 years | 15-03 | 41.56 ± 8.89 (6) | 5.27 ± 4.48 (6) | 17.72 ± 10.18 (6) | 315.44 ± 11.68 (6) |
| 15 years | 15A | 74.39 ± na (1) | 4.68 ± na (1) | 29.4 ± 13.11 (4) | 483.09 ± 22.32 (4) |
| 15 years | 15B | 33.54 ± na (1) | 3.3 ± na (1) | na | 389.08 ± 150.20 (4) |
| **15 years mean** |  | **44.7 ± 17.25 (20)d** | **4.59 ± 2.99 (20)c** | **17.28 ± 8.75 (19)d** | **384.34 ± 57.43 (20)ab** |
| 25 years | 25-01 | 137.84 ± 32.9 (6) | 14.56 ± 5.99 (6) | 9.89 ± 12.03 (6) | 312.77 ± 16.1 (6) |
| 25 years | 25-02 | 113.11 ± 23.98 (6) | 17.77 ± 12.01 (6) | 8.94 ± 9.41 (6) | 314.74 ± 38.09 (6) |
| 25 years | 25-03 | 84.65 ± 25.61 (6) | 9.56 ± 6.06 (6) | 13.45 ± 6.24 (6) | 249.68 ± 40.77 (6) |
| 25 years | 25A | 120.88 ± 24.49 (6) | 7.5 ± 1.67 (6) | na | 254.58 ± 80.21 (4) |
| 25 years | 25B | 155.99 ± 82.22 (6) | 10.38 ± 5.82 (6) | na | 253.79 ± 119.05 (4) |
| **25 years mean** |  | **114.52 ± 33.99 (20)e** | **13.46 ± 8.4 (20)d** | **10.76 ± 9.17 (18)c** | **285.45 ± 41.66 (20)a** |
| Protected | PFA | 93.49 ± 34.72 (6) | 21.57 ± 5.04 (6) | 25.33 ± 22.35 (6) | 412.03 ± 72.8 (6) |
| Protected | PFB | 115.85 ± 62.47 (6) | 19.16 ± 9.4 (6) | 19.21 ± 9.14 (6) | 484.55 ± 58.84 (6) |
| Protected | PFC | 134.02 ± 53.13 (6) | 24.03 ± 18.78 (6) | 15.4 ± 7.07 (6) | 526.07 ± 64.6 (6) |
| **Protected mean** |  | **114.45 ± 51.22 (18)e** | **21.58 ± 11.89 (18)d** | **19.98 ± 14.28 (18)bd** | **474.22 ± 75.71 (18)b** |
| **Grand total** |  | **54.23 ± 53.77 (114)** | **7.46 ± 9.68 (114)** | **20.92 ± 17.4 (108)** | **354.1 ± 96.43 (114)** |
| ***p - value*** |  | **χ^2^(5) = 88.752, *p* < 0.001** | **χ^2^(5) = 87.991, *p* < 0.001** | **χ^2^(5) = 53.556, *p* < 0.001** | **F_(5,54)_= 6.697, *p*<0.001** |

**Table S4.** Allometric equations used to determine tree mass and carbon pools of major mangrove species encountered in the study areas

| Species | AGB / Tree (kg) | Reference | BGB / Root (kg) | Reference |
| --- | --- | --- | --- | --- |
| *Avicennia marina;*  *Avicennia alba* | AGB = 0.308*D^2.11^ | ^[[6]](#endnote-6)^ | BGB = 1.28*DBH^1.17^ | 6 |
| *Bruguiera gymnorrhiza;*  *Bruguiera sexangula;*  *Bruguiera cylindrical* | Wood volume (Wv):  Wv = 0.0000754*(DBH^2.5^) | ^[[7]](#endnote-7)^ | BGB = 0.0188*DBH^2^*(DBH/(0.025DBH + 0.583))^0.909^ | 7 |
|  | Leaf biomass (Lb):  Lb = 10^(-1.1679+(1.4914*(LOG(DBH))))^ |  |  |  |
|  | Wood biomass (Wb):  Wb = Wv*ρ*1000  AGB = Lb + Wb |  |  |  |
| *Bruguiera parviflora* | AGB = 0.168*DBH^2.42^ | ^[[8]](#endnote-8)^ | BGB = 0.0188*DBH^2^*(DBH/(0.025DBH + 0.583))^0.909^ | 7 |
| *Rhizophora spp.* | Wood volume (Wv):  Wv = 0.0000695*DBH^2.64^ | 7 | BGB = 0.00698*DBH^2.61^ | ^[[9]](#endnote-9)^ |
|  | Leaf biomass (Lb):  Lb = 10^(-1.8571+(2.1072*(LOG(DBH))))^ |  |  |  |
|  | Wood biomass (Wb):  Wb = Wv*ρ*1000  Prop root biomass (PRb):  DBH≤5 cm PRb = Wb*0.101  DBH>5.0≤ 10 cm PRb = Wb*0.204  DBH>10≤ 15.0 cm PRb = Wb*0.356  DBH>15≤ 20.0 cm PRb = Wb*0.273  DBH>20 cm PRb = Wb*0.210  AGB = Lb + Wb + PRb |  |  |  |
| *Sonneratia alba* | Wood volume (Wv):  Wv = 0.0003841*DBH^2.10^ | 7 | BGB = 0.199*ρ^0.899^*DBH^2.22^ | ^[[10]](#endnote-10)^ |
|  | Leaf biomass (Lb):  Lb = 10^(-1.1679+(1.4914*(LOG(DBH))))^ |  |  |  |
|  | Wood biomass (Wb):  Wm = Wv*ρ*1000  AGB = Lb + Wb |  |  |  |
| *Xylocarpus granatum* | AGB = 0.0823*DBH^2.59^ | ^[[11]](#endnote-11)^ | BGB = 0.199*ρ^0.899^*DBH^2.22^ | 11 |
| Other species | AGB = 0.251*ρ*DBH^2.46^ | 11 | BGB = 0.199*ρ^0.899^*DBH^2.22^ | 11 |

Note: AGB = Aboveground biomass (kg); BGB = Belowground biomass (kg); DBH = Diameter at breast height (cm); ρ = wood density (g cm^-3^)

**Table S5.** Summary of sediment accretion and carbon burial calculations in sites 5 and 15 years post-harvest, assessed using ^210^Pb radionuclide.

Note: Data for the Protected Fringe and Interior sites are not provided because they are included in a previous publication ^14^.

|  | **Depth**  **(cm)** | **Bulk density (g/cm^3^)** | **Sedimenta**  **tion rates (g/cm^2^/yr)** | **Surface accretion rates (mm/yr)** | **210Pb activity (pCi/g)** | **Excess 210Pb Inventory (dpm/g)** | **Sediment age** | **Mass (g/cm^2^)** | **%C** | **C density**  **(g C/cm^3^)** | **Carbon burial (g C/m^2^/yr)** | **Carbon burial (Mg C/ha/yr)** |
| --- | --- | --- | --- | --- | --- | --- | --- | --- | --- | --- | --- | --- |
| **15-yr Fringe mangrove** | | | | | | | | | | | | |
|  | 0-2 | 0.40 | 0.44 | 7.82 | 0.65 |  | 2011.87 | 1.13 | 18.70 | 74.59 | 583.54 | 5.84 |
|  | 2-4 | 0.35 | 0.55 | 11.15 | 0.57 |  | 2010.08 | 0.99 | 18.70 | 65.81 | 733.61 | 7.34 |
|  | 4-6 | 0.66 | 0.31 | 3.32 | 0.73 |  | 2004.05 | 1.87 | 18.70 | 123.82 | 411.15 | 4.11 |
|  | 6-8 | 0.34 | 0.28 | 5.80 | 0.70 |  | 2000.60 | 0.97 | 18.70 | 64.35 | 372.93 | 3.73 |
|  | 8-10 | 0.29 | 0.47 | 11.52 | 0.50 |  | 1998.87 | 0.81 | 18.70 | 53.62 | 617.59 | 6.18 |
|  | 10-12 | 0.33 | 0.31 | 6.69 | 0.60 |  | 1995.88 | 0.94 | 18.70 | 61.91 | 414.01 | 4.14 |
|  | 12-14 | 0.30 | 0.29 | 6.73 | 0.59 |  | 1992.91 | 0.85 | 18.70 | 56.55 | 380.84 | 3.81 |
|  | 14-16 | 0.34 | 0.23 | 4.75 | 0.64 |  | 1988.70 | 0.96 | 18.45 | 62.56 | 297.44 | 2.97 |
|  | 16-18 | 0.19 | 0.30 | 10.86 | 0.52 |  | 1986.86 | 0.54 | 18.21 | 35.15 | 381.77 | 3.82 |
|  | 18-20 | 0.20 | 0.32 | 11.09 | 0.49 |  | 1985.05 | 0.57 | 18.21 | 37.05 | 410.92 | 4.11 |
|  | 20-24 | 0.38 | 0.13 | 2.36 | 0.70 |  | 1968.10 | 2.16 | 18.21 | 69.82 | 164.74 | 1.65 |
|  | 24-28 | 0.21 | 0.11 | 3.65 | 0.59 |  | 1957.15 | 1.19 | 18.21 | 38.47 | 140.53 | 1.41 |
|  | 28-32 | 0.26 | 0.07 | 1.78 | 0.59 |  | 1934.73 | 1.47 | 17.22 | 44.90 | 80.09 | 0.80 |
|  | 32-36 | 0.26 | 0.10 | 2.77 | 0.37 |  | 1920.30 | 1.45 | 16.22 | 41.67 | 115.50 | 1.16 |
| **Average** |  | **0.32** | **0.28** | **6.45** |  | **13.479** |  | **1.14** | **18.26** | **59.30** | **364.62** | **3.65** |
| **Stdev** |  | **0.12** |  | **3.55** |  |  |  | **0.46** | **0.71** | **22.64** | **194.77** | **1.95** |
| **15-yr Interior mangrove** | | | | | | | | | | | | |
|  | 0-2 | 0.24 | 0.62 | 18.44 | 0.54 |  | 2013.34 | 0.68 | 9.13 | 21.90 | 403.97 | 4.04 |
|  | 2-4 | 0.23 | 0.39 | 12.13 | 0.67 |  | 2011.69 | 0.64 | 9.13 | 20.71 | 251.18 | 2.51 |
|  | 4-6 | 0.33 | 0.51 | 10.94 | 0.57 |  | 2009.87 | 0.93 | 9.13 | 30.00 | 328.13 | 3.28 |
|  | 6-8 | 0.21 | 0.26 | 8.60 | 0.73 |  | 2007.54 | 0.60 | 9.13 | 19.52 | 167.92 | 1.68 |
|  | 8-10 | 0.25 | 0.52 | 14.44 | 0.54 |  | 2006.15 | 0.71 | 9.13 | 23.09 | 333.43 | 3.33 |
|  | 10-12 | 0.44 | 0.25 | 4.05 | 0.73 |  | 2001.22 | 1.24 | 9.13 | 40.00 | 162.02 | 1.62 |
|  | 12-14 | 0.39 | 0.36 | 6.56 | 0.57 |  | 1998.17 | 1.09 | 9.13 | 35.24 | 231.17 | 2.31 |
|  | 14-16 | 0.40 | 0.44 | 7.85 | 0.50 |  | 1995.62 | 1.13 | 11.59 | 46.25 | 363.07 | 3.63 |
|  | 16-18 | 0.21 | 0.18 | 5.91 | 0.74 |  | 1992.23 | 0.60 | 14.06 | 30.06 | 177.49 | 1.77 |
|  | 18-20 | 0.34 | 0.19 | 3.94 | 0.67 |  | 1987.16 | 0.97 | 14.06 | 48.23 | 190.28 | 1.90 |
|  | 20-24 | 0.32 | 0.31 | 6.85 | 0.50 |  | 1981.33 | 1.80 | 14.06 | 44.90 | 307.74 | 3.08 |
|  | 24-28 | 0.21 | 0.35 | 11.90 | 0.46 |  | 1977.97 | 1.16 | 14.06 | 28.96 | 344.44 | 3.44 |
|  | 28-32 | 0.41 | 0.24 | 4.15 | 0.30 |  | 1968.33 | 2.30 | 14.09 | 57.33 | 238.05 | 2.38 |
|  | 32-36 | 0.42 | 0.36 | 5.98 | 0.40 |  | 1961.64 | 2.39 | 14.13 | 59.68 | 356.77 | 3.57 |
|  | 36-40 | 0.47 | 0.15 | 2.27 | 0.46 |  | 1944.06 | 2.67 | 14.13 | 66.87 | 152.10 | 1.52 |
| **Average** |  | **0.32** | **0.34** | **8.27** |  | **10.284** |  | **1.26** | **11.61** | **38.18** | **267.18** | **2.67** |
| **Stdev** |  | **0.09** | **0.14** | **4.49** |  |  |  | **0.70** | **2.48** | **15.21** | **85.43** | **0.85** |
| **5-yr Fringe mangrove** | | | | | | | | | | | | |
|  | 0-2 | 0.43 | 0.58 | 9.59 | 0.82 |  | 2012.34 | 1.20 | 4.27 | 18.17 | 174.16 | 1.74 |
|  | 2-4 | 0.50 | 0.89 | 12.70 | 0.69 |  | 2010.77 | 1.40 | 4.27 | 21.18 | 268.95 | 2.69 |
|  | 4-6 | 0.37 | 0.52 | 9.86 | 0.82 |  | 2008.74 | 1.05 | 4.27 | 15.83 | 156.03 | 1.56 |
|  | 6-8 | 0.38 | 0.31 | 5.73 | 0.99 |  | 2005.25 | 1.07 | 4.27 | 16.16 | 92.62 | 0.93 |
|  | 8-10 | 0.36 | 0.66 | 12.85 | 0.78 |  | 2003.69 | 1.02 | 4.27 | 15.49 | 199.07 | 1.99 |
|  | 10-12 | 0.55 | 0.41 | 5.27 | 0.81 |  | 1999.89 | 1.56 | 4.27 | 23.63 | 124.42 | 1.24 |
|  | 12-14 | 0.59 | 0.26 | 3.11 | 0.92 |  | 1993.45 | 1.68 | 4.27 | 25.41 | 78.93 | 0.79 |
|  | 14-16 | 0.68 | 0.34 | 3.49 | 0.77 |  | 1987.72 | 1.92 | 4.14 | 28.16 | 98.20 | 0.98 |
|  | 16-18 | 0.59 | 0.29 | 3.47 | 0.76 |  | 1981.95 | 1.67 | 4.00 | 23.68 | 82.20 | 0.82 |
|  | 18-20 | 0.67 | 0.26 | 2.77 | 0.73 |  | 1974.74 | 1.90 | 4.00 | 26.92 | 74.65 | 0.75 |
|  | 20-24 | 0.69 | 0.82 | 8.40 | 0.56 |  | 1969.98 | 3.92 | 4.00 | 27.80 | 233.56 | 2.34 |
|  | 24-28 | 0.50 | 0.15 | 2.09 | 0.74 |  | 1950.81 | 2.80 | 4.00 | 19.82 | 41.35 | 0.41 |
|  | 28-32 | 0.38 | 0.19 | 3.47 | 0.61 |  | 1939.26 | 2.16 | 3.21 | 12.32 | 42.68 | 0.43 |
|  | 32-36 | 0.27 | 0.10 | 2.71 | 0.64 |  | 1924.50 | 1.52 | 2.42 | 6.51 | 17.64 | 0.18 |
|  | 36-40 | 0.17 | 0.05 | 2.05 | 0.67 |  | 1904.98 | 0.99 | 2.42 | 4.24 | 8.68 | 0.09 |
| **Average** |  | **0.48** | **0.39** | **5.84** |  | **13.547** |  | **1.72** | **3.88** | **19.02** | **112.88** | **1.13** |
| **Stdev** |  | **0.16** | **0.25** | **3.83** |  |  |  | **0.79** | **0.65** | **7.39** | **78.49** | **0.78** |
| **5-yr Interior mangrove** | | | | | | | | | | | | |
|  | 0-2 | 0.65 | 0.57 | 6.21 | 0.77 |  | 2011.88 | 1.83 | 5.16 | 33.36 | 207.11 | 2.07 |
|  | 2-4 | 0.75 | 0.52 | 4.94 | 0.80 |  | 2008.36 | 2.11 | 5.16 | 38.47 | 190.13 | 1.90 |
|  | 4-6 | 0.77 | 0.54 | 4.97 | 0.76 |  | 2004.75 | 2.16 | 5.16 | 39.54 | 196.72 | 1.97 |
|  | 6-8 | 0.64 | 0.44 | 4.81 | 0.73 |  | 2001.67 | 1.81 | 5.16 | 33.09 | 159.32 | 1.59 |
|  | 8-10 | 0.74 | 0.74 | 7.06 | 0.49 |  | 1998.30 | 2.09 | 5.16 | 38.20 | 269.54 | 2.70 |
|  | 10-12 | 0.54 | 0.26 | 3.43 | 0.75 |  | 1994.91 | 1.52 | 5.16 | 27.71 | 94.93 | 0.95 |
|  | 12-14 | 0.31 | 0.18 | 4.06 | 0.67 |  | 1993.29 | 0.88 | 5.16 | 16.14 | 65.57 | 0.66 |
|  | 14-16 | 0.36 | 0.64 | 12.72 | 0.54 |  | 1992.41 | 1.01 | 5.79 | 20.67 | 262.90 | 2.63 |
|  | 16-18 | 0.45 | 0.46 | 7.31 | 0.57 |  | 1990.93 | 1.27 | 6.41 | 28.77 | 210.37 | 2.10 |
|  | 18-20 | 0.52 | 0.17 | 2.35 | 0.71 |  | 1987.29 | 1.47 | 6.41 | 33.45 | 78.44 | 0.78 |
|  | 20-24 | 0.41 | 0.19 | 3.23 | 0.72 |  | 1980.48 | 2.29 | 6.41 | 26.01 | 84.09 | 0.84 |
|  | 24-28 | 0.40 | 0.13 | 2.34 | 0.95 |  | 1963.41 | 2.25 | 6.41 | 25.59 | 59.94 | 0.60 |
|  | 28-32 | 0.55 | 6.19 | 79.28 | 0.43 |  | 1962.90 | 3.12 | 6.72 | 37.14 | 2944.59 | 29.45 |
|  | 32-36 | 0.74 | 0.29 | 2.83 | 0.57 |  | 1948.76 | 4.15 | 7.02 | 51.65 | 146.14 | 1.46 |
|  | 36-40 | 0.63 | 0.23 | 2.58 | 0.54 |  | 1933.25 | 3.54 | 7.02 | 44.05 | 113.54 | 1.14 |
| **Average** |  | **0.56** | **0.77** | **9.87** | **0.67** | **18.394** |  | **2.10** | **5.89** | **32.92** | **338.89** | **3.39** |
| **Stdev** |  | **0.15** | **1.51** | **19.39** | **0.14** |  |  | **0.91** | **0.76** | **9.15** | **724.15** | **7.24** |

**References**

1. . Hansen, M. C., et al. High-resolution global maps of 21st-century forest cover change. *Science* 342.6160: 850-853 (2013). [↑](#endnote-ref-1)
2. . PT. Bintuni Utama Murni Wood Industries (BUMWI), Annual Harvest Production Report for the 2001–2018 period in PT. BUMWI Concession. Internal Report (2020). [↑](#endnote-ref-2)
3. . PT. Bintuni Utama Murni Wood Industries (BUMWI), Reports of Draught Surveys: 2015–Mid. 2016. Indonesia (2016). [↑](#endnote-ref-3)
4. . PT. Sucofindo, Provisional Reports of Draught Surveys – PT. BUMWI: 2016–2018. Indonesia (2020a). [↑](#endnote-ref-4)
5. . PT. Sucofindo, Provisional Reports of Draught Surveys – PT. BUMWI: 2016–2018. Indonesia (2020b). [↑](#endnote-ref-5)
6. . Comley, B. W. T. A. & McGuinness, K. A. A. Above- and below-ground biomass, and allometry, of four common northern Australian mangroves. 431–436 (2005). [↑](#endnote-ref-6)
7. . Kauffman, J. B. & Cole, T. G. Micronesian Mangrove Forest Structure and Tree Responses to a Severe Typhoon. 1077–1084 (2010). doi:10.1007/s13157-010-0114-y [↑](#endnote-ref-7)
8. . Ong, J. E., Gong, W. K. & Wong, C. H. Allometry and partitioning of the mangrove, Rhizophora apiculata. *For. Ecol. Manage.* **188,** 395–408 (2004). [↑](#endnote-ref-8)
9. . Tamai, S., Nakasuga, T., Tabuchi, R. & Ogino, K. Standing biomass of mangrove forests in southern Thailand. *J. Japan For. Soc.* **68,** 384–388 (1983). [↑](#endnote-ref-9)
10. . Komiyama, A., Poungparn, S. & Kato, S. Common allometric equations for estimating the tree weight of mangroves. 471–477 (2005). doi:10.1017/S0266467405002476. [↑](#endnote-ref-10)
11. . Clough, B. F., Dixon, P. & Dalhaus, O. Allometric Relationships for Estimating Biomass in Multi-stemmed Mangrove Trees. *Aust. J. Bot.* **45,** 1023 (1997).

    12. Gaveau, D.L.A., Salim, M.A. & Husnayaen. Papua Atlas. https://atlas.cifor.org/papua/ (2019).

    13. Ministry of Forestry, Republic of Indonesia. Boundary establishment of PT. Bintuni Utama Murni Wood Industries for 78669.29 ha in Bintuni Bay Regency, West Papua Province. Jakarta: Republic of Indonesia (2013).

    14. Sasmito, S. D. et al. Organic carbon burial and sources in soils of coastal mudflat and mangrove ecosystems. Catena 187, 104414 (2020). [↑](#endnote-ref-11)
